# Ecological drivers of nesting behavior in a subtropical city: An observational study on spotted doves

**DOI:** 10.1002/ece3.11655

**Published:** 2024-07-03

**Authors:** Yao Sheng, Mengjie Lu, Junpeng Bai, Xiaobin Xie, Long Ma, Wanyou Li, Zhen Zhang, Fang Ming, Xueli Zhang, Ziwei Zhang, Zhifeng Xu, Yuqing Han, Bicai Guan, Luzhang Ruan

**Affiliations:** ^1^ School of Life Sciences, State Ministry of Education Key Laboratory of Poyang Lake Environment and Resource Utilization Nanchang University Nanchang China; ^2^ Qingdao Jiaodong International Airport Qingdao China; ^3^ Jinhui Liquor Company Limited Longnan China; ^4^ Guangdong Maoming Health Vocational College Maoming China

**Keywords:** habitat selection, nest reuse, predation, reproductive success, urban habitat

## Abstract

Due to rapid homogenization in habitat types as a result of urbanization, some urban birds adapt their nesting strategies to changes in local habitat characteristics. Bird nesting decisions might have been mainly linked to resource constraints and ensuring reproductive success. In this study, we examined patterns of nesting behavior by spotted doves (*Spilopelia chinensis*) in a rapidly urbanizing area of Nanchang, China using ArcGIS 10.8, satellite tracking, camera traps, and field survey. To explore the mechanisms underlying nesting behavior in urban habitats, we assessed the correlations between nest reuse and reproductive success, and between nest reuse and nest predation. From December 2018 to December 2021, a total of 302 breeding nests were surveyed. The results revealed that the nest reuse rate was 38.08% (*n* = 115). Nests closer to trunk, with lower nest position and higher large‐scale urbanization score tended to have higher reuse rate. In addition, nests with the higher the nest height and percent of canopy cover, and the lower small‐scale urbanization score were more likely to reproduce successfully, and the reused nests also reproduce more successfully. The reproductive success associated with nest reuse was significantly higher than that associated with new nests (*χ* ^2^ = 8.461, *p* = .004). High degree of urbanization promoted nest reuse of spotted doves (large‐scale urbanization score, *z* = 2.094, *p* = .036), which apparently enhanced their reproductive success (nest reuse, *z* = 2.737, *p* = .006). In conclusion, a nest structure with good permeability is the material basis for the nest reuse in spotted dove, while the relatively low risk of predation in urban habitat and the scarcity of nest site resources due to urbanization increase the tendency of birds to reuse old nests, which is associated with their reproductive success and evolutionary fitness.

## Graphical abstract

High degree of urbanization promoted nest reuse. Nest reuse enhances avian reproductive success. Nest reuse did not increase the risk of predators.

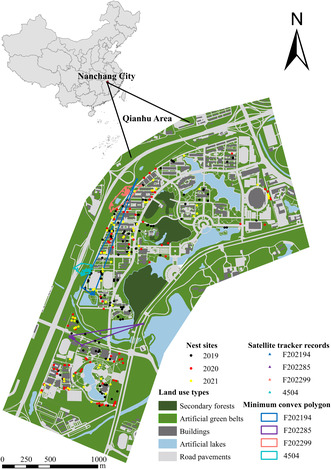

## 1 INTRODUCTION

Given the rapid pace of urban development, there is a need to identify the response mechanisms of model species' life history evolution in order to develop effective conservation strategies for urban landscapes (Lerman et al., 2021; Shriver et al., 2021). Reproduction is one of the energetically most expensive avian life‐history stages (Frommhold et al., 2019), and is, therefore, more responsive to environmental change. Different from natural habitats, urban habitats are a result of anthropogenic‐induced processes of rapid habitat change, which accelerates phenotypic differentiation and can directly affect reproductive effort and success of birds (Corsini et al., 2021; Sepp et al., 2018). Birds often adapt their breeding strategies to changes in local habitat characteristics to maximize reproductive success (Montreuil‐Spencer et al., 2019; Sepp et al., 2018; Tomasevic & Marzluff, 2020). Nesting is the beginning of a large energy expenditure during the breeding season, which is crucial for birds because this stage is significant for breeding success (Moreno et al., 2008). The extent of urbanization can lead to significant changes in habitat ecological factors, such as the availability of suitable nesting sites (Champness et al., 2019), local micro‐climate (Sullivan et al., 2021), the timing of resource phenology (Neil & Wu, 2006), the availability of food required for breeding (Palacio, 2020; Seress et al., 2020; Tryjanowski et al., 2015), compositions of heterospecific communities (e.g., predators, parasites, competing species; Juarez et al., 2020), and factors associated with human presence (e.g., noise, pollution, artificial light; Francis et al., 2011; Leveau, 2018; Mueller et al., 2020). Consequently, urban and non‐urban populations of the same species usually differ significantly in nesting strategies and performance (Chamberlain et al., 2009).

In urbanized areas where food and suitable nesting sites are limited, reusing nests can be one of the breeding strategies of birds to reduce energy expenditure (Mainwaring & Hartley, 2013). Since the energetic cost of nesting for birds is usually high, the pre‐breeding costs can be significantly reduced if old nests are reused. Birds that reuse old nests can realize increased reproductive success by devoting more energy to later stages of the breeding cycle (Visser et al., 2019). Nest reuse in open‐nesting birds is relatively rare in the natural environment because such nests are more likely to be exposed to a variety of risks than other types of nests. For example, old nests may be more susceptible to ectoparasite infections, which can affect chick growth and survival (Brown & Brown, 1986; Tomas et al., 2007), as well as predators may also be more likely to memorize the location of the nest, thus increasing the risk of nest predation (Boves et al., 2013; Herzog et al., 2018). Despite this, some urban open‐nesting birds still choose to reuse their old nests for breeding, inevitably because nest reuse can be more beneficial and is a good breeding strategy for them to adapt to urban habitats, but this needs to be confirmed by further research. In addition, most of the current studies on nest reuse have been conducted in natural environments, but the impact of urban habitats cannot be ignored.

We consider the spotted dove to be a suitable model species for studying the effects of nesting behavior in urban environments for the following two reasons: (1) their nests can be maintained for a long time after breeding has ended, and could be continuously reused as it is delicately constructed mainly from branches, which provide good stability and permeability, and (2) they are an annual multiple‐brooded species. Therefore, the aim of this study was to explore adaptive strategies of nesting behavior on spotted doves in urban habitats. In the current observational study of one city population of spotted doves, we report aspects of nesting behavior (new nest versus reuse of old nest) in association with breeding success (with versus without fledglings) and 14 characteristics of the breeding environment. Specifically, we will address the following questions: (1) spotted doves would prefer to reuse nests for breeding in urban habitats and (2) breeding success would be higher for reused nests than for newly constructed nests.

## 2 METHODS

### 2.1 Study area and species

**FIGURE 1:**
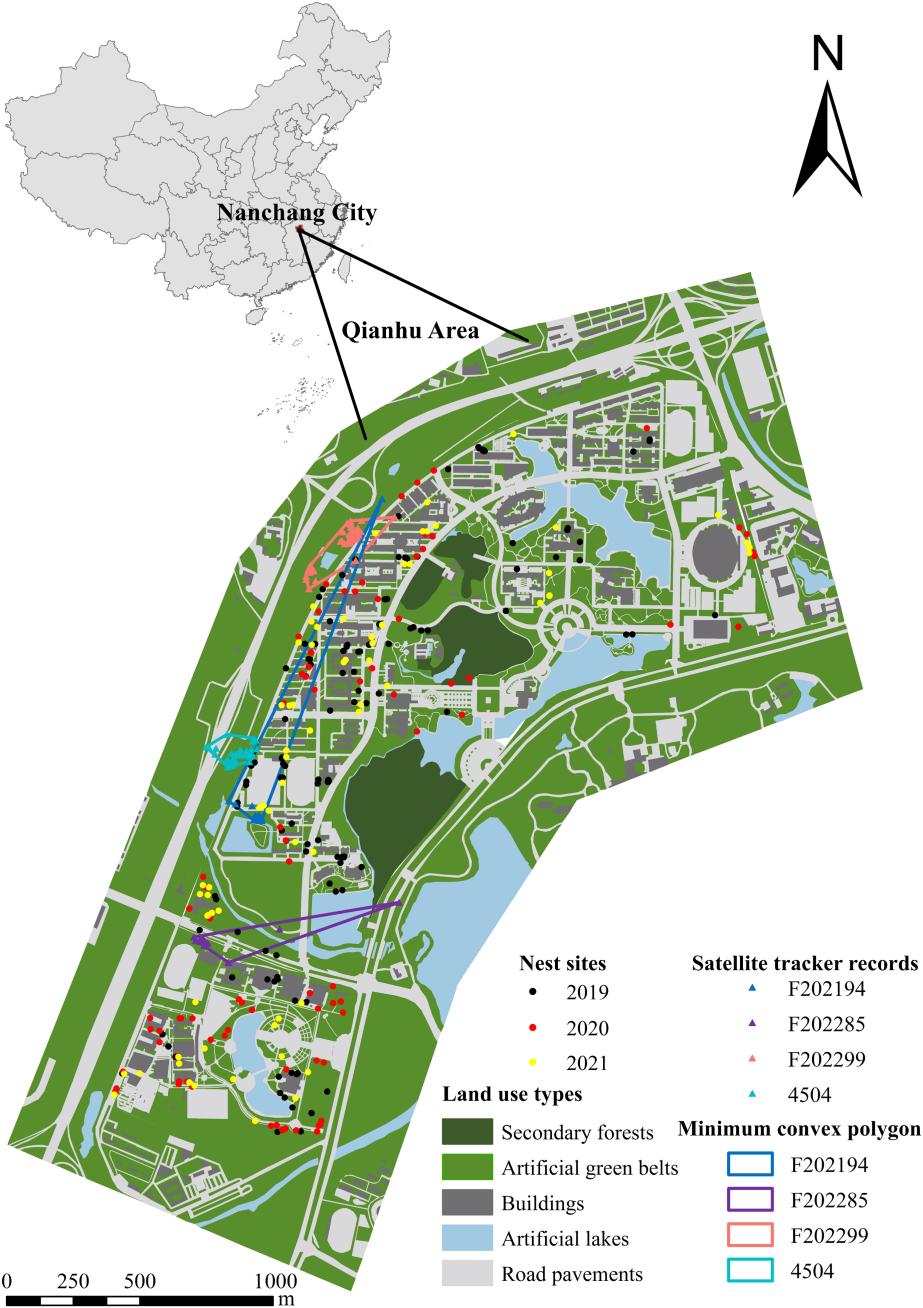
Nest‐site locations of spotted doves and land use types of study area in Nanchang, China. A total of 302 nest sites were monitored from December 2018 to December 2021. Black, red, and yellow‐filled circles denote separate nest sites in 2019 (*n* = 145), 2020 (*n* = 87), and 2021 (*n* = 70). Blue triangles represent GPS position records from satellite trackers (Nos.: F202299, F202194, 4504, and F202285) on four spotted doves, and the red line represents the minimum convex polygon containing all points.

This study was conducted in Nanchang City, Jiangxi Province, China. The study area covers 300 ha (115°47′12″–115°38′34″ E, 28°39′4″–28°40′16″ N) and it has about 55,000 inhabitants (Figure 1). In the study location, the habitat type was artificial. However, no nest was recorded on buildings, which may be due to human interference. Therefore, nest sites on buildings were not discussed here. The Camphor tree (*Cinnamomum camphora*) and Deodar cedar (*Cedrus deodara*) were the two most common tree species in the survey area. According to the differences in building indexes and vegetation categories of urban habitat, the land use types within the study area include road pavements, artificial green belts, artificial lakes, buildings, and secondary forests (Han et al., 2019). The spotted dove is a common urban open‐nesting species in East Asia, with a stable clutch size of two eggs, an incubation period averaging 18 days, and chicks fledging after 17–20 days (Zhou et al., 2004). The nest resembles a humble flat plate. Herbaceous rhizomes make up most of its upper layer, while decaying tree branches make up most of its lower layer (Gao et al., 2018; Zhao, 2001).

### 2.2 Data collection

Due to our extensive experience in the field, we could easily distinguish the nests of spotted dove from other species visually. Moreover, its nest was the most rudimentary in the study area resembling a flat plate, and could be easily distinguish from the nests of other species. Reused nests of spotted dove are thicker and have a distinctive layering of old and new nest material that can be clearly recognized by the naked eye. After identifying the new and reused nests, we also checked whether spotted doves were using the nests for breeding. In December 2018, the study area was thoroughly surveyed for spotted dove nests labeled as nests bred during the previous breeding season. There's no way to classify the 2018 nests as new or old nests, so the breeding data in 2018 were excluded from the data pool for further analysis. From January 2019 to December 2021, all new spotted dove nests were searched once a week. Once a new nest was located, its location was marked using the Google Earth software program (Version 7.3.2, Google, 2019). A bird breeding in an existing old nest was defined as nest reuse, whereas, a bird breeding in a newly constructed nest was classified as nesting (Jimenez‐Franco et al., 2014).

All occupied nests were continuously monitored once a week before the first egg was laid. After the first egg was laid, nests were visited every 3 days to confirm breeding status until juveniles fledged or the nest was depredated, and to determine whether the nest was predated or contained ectoparasites (Mayfield, 1975). Here, we defined reproductive duration as the number of days from first egg laying to breeding success or failure, and defined at least one fledgling leaving the nest as breeding success while no fledging leaving the nest as breeding failure (Bensouilah & Barrientos, 2021). When the laying date of the first egg was not known precisely, it was estimated by backcasting the hatching date of the first bird (Newton & Moss, 1986). In order to monitor predators without disturbing breeding birds, passive infrared camera traps (Version H982, Shenzhen, China) were placed at least 50 cm away from the nest after egg laying.

We monitored a total of 302 breeding nests in this study. However, due to the COVID‐19 pandemic that occurred during this study, we completed continuous monitoring of only 91 breeding nests, while the remaining 211 breeding nests (for which continuous monitoring was not completed) were monitored based on the experience of the previous 91 nests to determine breeding success. Based on the monitoring experience of these 91 nests, we developed rules to distinguish between breeding successful nests with visible feces and failed nests with few feces. Since the parents only clean the feces until the chicks are 6 days old, there must have been a large accumulation of feces in the nest after that. Therefore, the amount of feces can be a very useful indicator of whether the condition of the nestling has been present. Therefore, we can conclude that the absence of feces in an empty nest is considered a breeding failure, while visible feces in an empty nest is considered a breeding success (Figure A: Appendix S1).

To avoid excessive disturbance to the birds, a total of 14 ecological factors were recorded after breeding attempts ceased according to the methods described by Yeldell et al. (2017), Wendt and Johnson (2017), and Han et al. (2019), including factors about nesting tree (tree species, tree height, nest height, ground diameter, diameter of the tree canopy), nest concealment (distance from tree edge, the percentage of the canopy cover, percentage of the canopy cover above the nest, concealment), nest stability (branch support, nest position), urbanization degree (small‐scale urbanization score, large‐scale urbanization score) and food accessibility (the distance to the water sources) (Table A: Appendix S1). For the measurement of nest position, we record with 1 if the nest is located on the primary branch, with 2 on the secondary branch, with 3 on the tertiary branch, and so on. Using images of the canopy and the canopy above the nest taken with a Canon camera (Version EOS R6, Tokyo, Japan), the percentage of the canopy cover and the percentage of the canopy cover above the nest were respectively calculated as a percentage of the opaque area. Concealment was calculated utilizing images that covered all four directions from the nest within 1 m. When the distance to the water sources was more than what could be estimated using the tape‐measuring with a measuring range of 30 m, Google Earth was utilized to determine the distance to artificial lakes or ponds. All of the factors were described in detail in Table A: Appendix S1.

The study area is highly urbanized, but parts of it are patches of secondary forest, which means that there may be differences in the degree of urbanization at each nest site. The urbanization score is necessary in studies related to urbanization. This led to the calculation of the urbanization score for each nest site in the research area. By combining the methods described by Liker et al. (2008), Seress et al. (2014), and Saccavino et al. (2018), the degree of urbanization was measured using two separate scales: small‐scale urbanization score and large‐scale urbanization score. According to Saccavino et al. (2018), relevant factors for birds were used to determine the degree of small‐scale urbanization at nest sites, including the height of the highest building as determined by the number of floors, building percentages, vegetation percentages, impervious surfaces percentages, and the distance from the nest site to the nearest nighttime luminous source. For the height of the highest building, building percentage, vegetation percentage, and impervious surfaces percentage, the circular sample area was examined with the bird's nest site as the center and the minimum nest spacing as the diameter. According to our monitoring, the minimum distance between nests of breeding spotted doves at the same time was 60 m. Therefore, we set 60 m as the minimum nest spacing of spotted doves. ArcGIS 10.8 geoinformatics software was used to provide accurate quantitative measurements of the land‐cover features of each nest site. All landscape object polygons were identified, traced, and classified as either vegetation, buildings, impervious surfaces, or water bodies. After that, by summing the areas of the polygons belonging to each of the four categories, the total percentage area of each cover type was determined. The distance from the nest site to nighttime luminous source was measured directly with a tape‐measure; when the distance was too great to measure with tape measure, GPS measurements on Google Earth were taken. The small‐scale urbanization score was then derived for each nest site using the PC1 score from a PCA of the five factors mentioned above. Two principal components were identified using the PCA on the small‐scale urbanization characteristics variables, which together accounted for 93.49% of the variation (Table G: Appendix S1).

During the breeding period, the birds need to leave the nest for forage, and the fact that birds have a high dispersal capacity means that environmental resources in the wider area around the nest site are also very important. We therefore examine a circular sample area centered on the nest location and with the maximum flight distance of the bird (see below) as the diameter to determine the large‐scale urbanization score of the nest site. Building percentage, vegetation percentage, and impervious surfaces percentage were then determined (Liker et al., 2008). The large‐scale urbanization score was calculated using the PC1 (83.95%) of these three characteristics (Table G: Appendix S1). Four spotted doves were caught using mist nets around their nests during breeding seasons in 2019 and 2021 in order to deploy backpack satellite trackers with solar cells to maintain battery power in order to determine the maximum flight distance of the spotted dove (Table F: Appendix S1). The four spotted doves, each weighing more than 180 g, were fitted with satellite trackers from Hangzhou Simo Technology Co., Ltd, China (S08, weight 8.4 g). The satellite trackers used the Global Positioning System (GPS) to gather location data, and then they used a mobile communications network to send the data back to a server. In this study, considering the power consumption, the satellite tracker collected one GPS position per day. Using ArcGIS 10.8, the maximum flight distance was calculated as the diameter of a circular area. The ones with F202299 were male, and those with F202194, 4504, and F202285 were female. The satellite trackers 4504, F202299, F202194, and F202285 continued to return data for 32, 115, 201, and 48 days, respectively. A total of 188 positions of four spotted doves were recorded using GPS. The maximum home range of the spotted dove ranged from 15,265.30 to 91,145.80 m^2^. The maximum and minimum flight distance range of the spotted dove ranged from 144.12 to 421.23 m and 0.22 to 1.00 m, respectively (Table F: Appendix S1). Finally, based on a home range diameter of 421.23 m, building percentage, vegetation percentage, and impervious surfaces percentage of the circular samples were collected using ArcGIS 10.8. Two principal components were produced by the PCA on the large‐scale urbanization characteristics variables, and they accounted for 95.500% of the total variance (Table G: Appendix S1).

Nest material was sampled from intact nests for analysis and nest material parameters were measured after the field survey had ended. The following nest material parameters were gauged: mean twig diameter (cm), twig volume (cm^3^), mean vimen diameter (cm), vimen volume (cm^3^), and total volume of twig and vimen (cm^3^). Additionally, three nest size parameters were measured: nest diameter (cm), thickness (cm), and depth (cm). Twig volume is calculated by measuring the length and diameter of each twig, and then calculating the volume of each twig by the formula *V* = *π ×* (*d*/2)^2^ × *h*, and the sum of the volumes of all twigs in this nest is twig volume. The volume of vimen volume can be obtained in the same way, and total volume of twig and vimen is equal to twig volume plus vimen volume.

### 2.3 Statistical analyses

Since the sample size of nests was > 30 (Central Limit Theorem), *t*‐test with the significance level set at 0.05 was used to assess reproductive success and failure (Lilliefors, 1967), as well as nest reuse and building (Table H, B, C: Appendix S1).

To evaluate the correlation between the ecological factors, a Pearson's rank correlation test was run (Tables D, E: Appendix S1; Maritz, 1995). Using nest reuse or nesting (0/1) as the dependent variable criteria, a logistic regression (Hastie, 2017) was used to predict the mechanism behind reproductive success and nest reuse as well as the influence of ecological factors on nest reuse. The most explanatory and uncorrelated variables were kept, while the others were eliminated based on the greatest correlation coefficients (Pearson's rank correlation, |*r*| > .50); (Inselman et al., 2015; Soh et al., 2002) in order to reduce over‐fitting brought on by redundant variables. Using a negative binomial regression model, the impact of the ecological factors (see below) on the spotted dove's reproductive duration was calculated. There were 12 variables total (nest height, diameter of the tree canopy, percent of canopy cover over the nest, branch support, nest position, distance from tree edge, percent of canopy cover, concealment, distance to water sources, small‐scale urbanization score, large‐scale urbanization score, and nest reuse). Before creating the models for any of the aforementioned models, all the data were standardized by R 4.1.2 (R Development Core Team, 2022). The Akaike Information Criterion (AIC_*c*_) corrected was then used to select the models, and the AIC_*c*_ difference (ΔAIC_*c*_) between the model with the lowest AIC_*c*_ value and all other models was calculated for each model (Melles et al., 2003). The best models in a set were those with the lowest ΔAIC_*c*_. The support for each model was also shown by Akaike weights (*w*_*i*_; Han et al., 2019). R 4.1.2 was used to perform all statistical analyses. Figures were created with OriginPro (Version 2022b, Northampton, MA, USA).

## 3 RESULTS

### 3.1 Nesting tree species and breeding outcome

**FIGURE 2:**
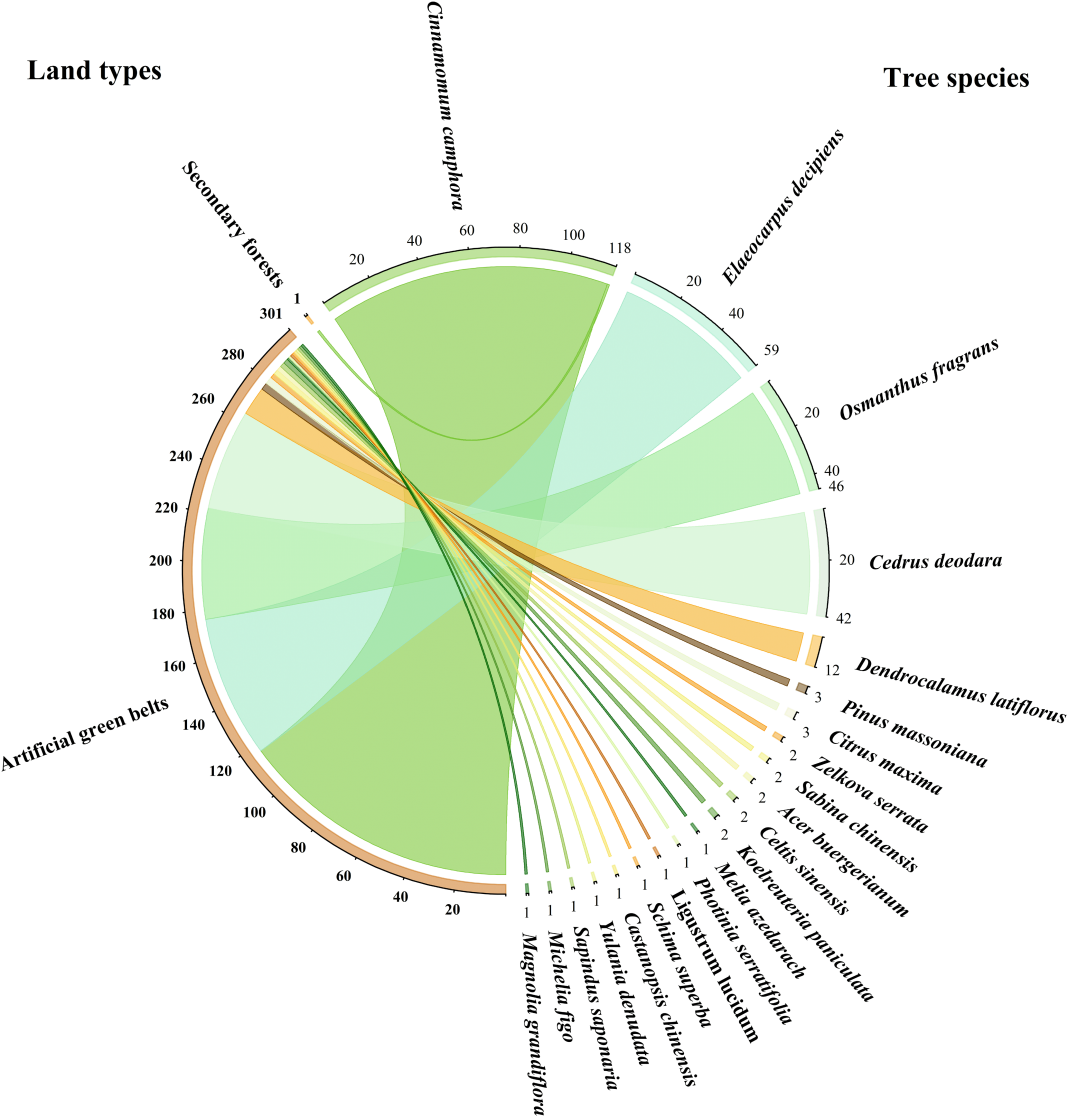
Total number of spotted dove' nests in different habitats and tree species during the study period.

In the study area, a total of 302 spotted dove nests were monitored (including 145 in 2019, 87 in 2020, and 70 in 2021). The majority of the nests (99.67%, *n* = 301) were discovered in trees situated in artificial green belts, while only one nest (0.37%) was discovered in a secondary forest. The highest number of spotted dove nests were observed in camphor tree *Cinnamomum camphora* (38.74%, *n* = 117), followed by bead tree *Elaeocarpus decipiens* (19.54%, *n* = 59) and sweet osmanthus *Osmanthus fragrans* (15.23%, *n* = 46; Figure 2).

Breeding outcome was determined for all nests, including 136 (45.03%) reproductively successful nests and 166 (54.97%) failed nests. Nest utilization was also determined, including 115 (38.08%) nest reuse and 187 (61.92%) nest building. Of the 115 nests classified as nest reuse, 64 were successful nests (55.65%) and 51 were failed nests (44.35%). Of the 187 nests classified as nest building, 72 were successful nests (38.50%) and 115 were failed nests (61.50%). The reproductive success associated with nest reuse was significantly higher than that associated with nest building (*χ* ^2^ = 8.461, *df* = 1, *p* = .004). A total of 91 breeding nests were monitored continuously, of which 36 (39.56%) were successful nests and 55 (60.44%) were failed nests. These 91 nests were also determined as 42 (46.15%) nest reuse and 49 (53.85%) nest building.

### 3.2 Nest predation

A total of 17 nest predators but no chick predators were detected by camera monitoring, including 12 aerial predators and 5 ground predators. The aerial predators were Eurasian jays (*Garrulus glandarius*, *n* = 5), red‐billed blue magpies (*Urocissa erythrorhyncha*, *n* = 5) and great tits (*Parus major*, *n* = 2), while the ground predators included the buff‐breasted rats (*Rattus flavipectus*, *n* = 3) and domestic cats (*Felis catus*, *n* = 2). A significantly higher number of aerial predators than ground predators was observed (*χ* ^2^ = 9.529, *df* = 1, *p* = .003). The 17 different nests that were preyed upon comprised 6 reused nests and 11 new nests. No significant difference in predation was found between nest reuse and nest building (*χ* ^2^ = 0.805, *df* = 1, *p* = .267).

### 3.3 Key factors affecting birds' nest reuse

Distance from tree edge (105.577 ± 94.316 cm) and large‐scale urbanization score (2.989 ± 19.560) of nest reuse were significantly higher than those of nest building (distance from tree edge = 83.604 ± 68.764 cm, *t* = 2.169, *df* = 300, *p* = .031; large‐scale urbanization score = −1.838 ± 15.910, *t* = 2.343, *df* = 300, *p* = .020). No significant difference was observed in the other factors between nest reuse and nest building (Table H: Appendix S1). Thickness (5.692 ± 2.558 cm), twig volume (5592.321 ± 6911.079 cm^3^), and mean vimen diameter (0.812 ± 0.207 cm) in reused nests were significantly smaller than those in new nests (thickness = 6.617 ± 2.680 cm, *t* = 2.227, *df* = 175, *p* = .027; twig volume = 10,269.928 ± 11,563.372 cm^3^, *t* = 2.059, *df* = 71, *p* = .043; mean vimen diameter = 0.900 ± 0.130 cm, *t* = 2.186, *df* = 71, *p* = .032; Table C: Appendix S1).

**FIGURE 3:**
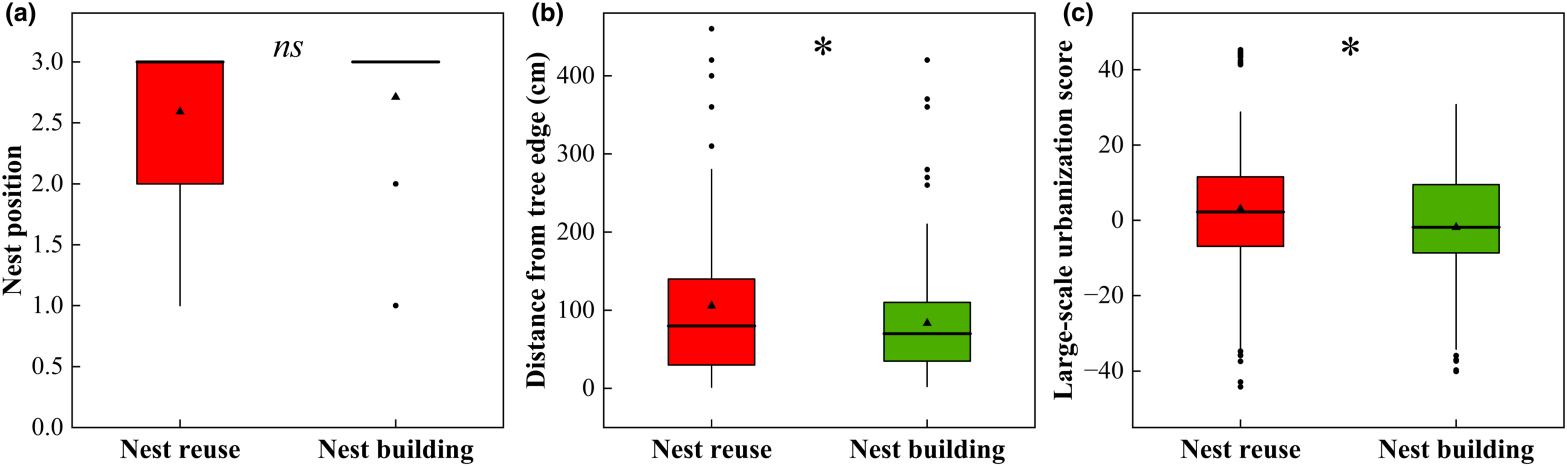
Mean ± SD of best model parameters affecting nest reuse and nesting of spotted doves. Bold lines denote median values, and boxes represent interquartile ranges (25%–75% percentiles). Plain lines show 1.5 × interquartile range, while filled circles and triangles represent separate outliers and mean values. ns (*p* > .050), * (*p* < .050). Variable descriptions are found in Table A: Appendix S1.

The best model (top‐rank model, ΔAIC_*c*_ = 0) for predicting nest reuse, according to the findings of a logistic regression, was [nest position + distance from tree edge + large‐scale urbanization score] (AIC_*c*_ = 396.070, *ω*_*i*_ = 0.420; Table 1; Figure 3). Spotted doves were more likely to reuse nests that are secluded (*z* = 1.979, *p* = .048), stable (*z* = −1.993, *p* = .046), and located in highly urbanized areas (*z* = 2.094, *p* = .036).

**TABLE 1:** Ecological factors that influence reproductive success and nest reuse in the spotted dove, examined by fitting generalized linear models. reproductive success (0/1) and nest reuse (0/1) as the dependent variable.

Models	Factors	Coefficients	SE	*z*	*p*
Nest reuse	Intercept	−0.505	0.122	−4.156	<.001***
	Distance from tree edge	0.240	0.121	1.979	.048*
	Nest position	−0.238	0.120	−1.993	.046*
	Large‐scale urbanization score	0.263	0.126	2.094	.036*
Reproductive success	Intercept	−0.497	0.164	−3.036	.002**
	Nest height	0.684	0.137	4.998	<.001***
	Percent of canopy cover	0.570	0.151	3.786	<.001***
	Small‐scale urbanization score	−0.265	0.131	−2.014	.044*
	Nest reuse	0.708	0.259	2.737	.006**

*Note*: Significant results (*p* < .05) were highlighted in bold. * (*p* < .050), ** (*p* < .010), *** (*p* < .001). Variable descriptions are found in Table A: Appendix S1.

Abbreviation: SE, standard error.

### 3.4 Key factors affecting birds' reproduction

Tree height (7.792 ± 2.375 m), nest height (6.283 ± 2.053 m), ground diameter (27.113 ± 11.455 cm), nest position (2.735 ± 0.505), and percent of canopy cover (81.360 ± 9.805%) of successful nests were significantly higher than those of the failed nests (tree height = 6.595 ± 2.186 m, *t* = 4.551, *df* = 300, *p* < .001; nest height = 5.097 ± 1.962 m, *t* = 5.118, *df* = 300, *p* < .001; ground diameter = 22.540 ± 10.216 cm, *t* = 3.664, *df* = 300, *p* < .001; nest position = 2.608 ± 0.601, *t* = 1.993, *df* = 300, *p* = .047; percent of canopy cover = 76.494 ± 15.872%, *t* = 3.263, *df* = 300, *p* = .001). No significant difference was observed in the other factors between reproductive success and reproductive failure nests (Table H: Appendix S1). In addition, the Nest diameter (16.967 ± 2.058 cm) of the reproductive success nests was significantly lower than that of the reproductive failure nests (nest diameter = 17.881 ± 2.344 cm, *t* = −2.660, *df* = 175, *p* = .009; Table B: Appendix S1).

**FIGURE 4:**
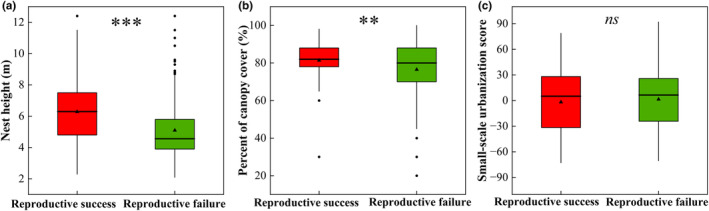
Mean ± SD of best model parameters affecting reproductive success and reproductive failure of spotted doves. Bold lines denote median values, and boxes represent interquartile ranges (25%–75% percentiles). Plain lines show 1.5 × interquartile range, while filled circles and triangles represent separate outliers and mean values. ns (*p* > .050), ** (*p* < .010), *** (*p* < .001). Variables descriptions are found in Table A: Appendix S1.

The best model (top‐rank model, ΔAIC_*c*_ = 0) for forecasting the reproductive success of the spotted dove, according to the findings of a logistic regression, was [nest height + percent of canopy cover + small‐scale urbanization score + nest reuse] (AIC_*c*_ = 375.390, *ω*_*i*_ = 0.716; Table 1). Nest height, percent canopy cover, and nest reuse were significantly correlated with reproductive success in this model (*z* = 4.998, *p* < .001; *z* = 3.786, *p* < .001; z = 2.737, *p =* .006), whereas small‐scale urbanization score (*z* = −2.014, *p* = .044) was significantly negatively correlated with reproductive success (Figure 4). Additionally, the factors that affect reproductive duration were examined. The findings of a negative binomial regression showed that the model [nest height + percent of canopy cover] was the top‐ranked model for forecasting the spotted dove's reproductive duration (AIC_*c*_ = 745.060, *ω*_*i*_ = 0.853; Table 2). In this model, reproductive duration was significantly positively correlated with nest height and percent of canopy cover (*t* = 2.312, *p* = .023; *t* = 3.827, *p* < .001; Figure B: Appendix S1). Additionally, a Pearson's rank correlation test revealed a negative association between the large‐scale urbanization score and the spotted dove's reproductive duration (Figure C: Appendix S1).

**TABLE 2:** Ecological factors that influence reproductive duration in the spotted dove, examined by negative binomial regression models. reproductive duration as the dependent variable.

Factors	Coefficients	SE	*t*	*p*
Intercept	3.036	0.065	46.901	<.001***
Nest height	0.152	0.066	2.312	.023*
Percent of canopy cover	0.255	0.067	3.827	<.001***

*Note*: Significant results (*p* < .05) were highlighted in bold. * (*p* < .050), *** (*p* < .001). Variable descriptions are found in Table A: Appendix S1.

Abbreviation: SE, standard error.

## 4 DISCUSSION

### 4.1 Potential causes of nesting behavior

High degree of urbanization may promote nest reuse, which is a reproductive strategy that birds trade‐off between the benefits and costs of degree of urbanization to achieve the greater lifetime reproductive success (Martin, 1995). It was found that 99.67% of the nests were located in artificial green belts. The fact that new and reused nests are not observed in the same ecological environments can be explained by resource constraints varying in space (Han et al., 2019). The lack of tall trees and intense anthropogenic disturbances associated with urbanization further limit the availability of suitable nest site resources (Tomasevic & Marzluff, 2017), thus promoting the nest reuse by urban spotted dove. Nnest reuse model analysis highlighted that large‐scale urbanization score was significantly positively correlated with nest reuse (Table 1). The results of the *t*‐test also highlighted that nest reuse has a higher large‐scale urbanization score (Table H: Appendix S1). Moreover, reused nests were closer to the main trunk of the tree and farther from tree edge than new nests (Table 1), indicating that reused nests are structurally more stable and have a relatively lower risk of reproductive failure. The modification of reproductive strategies, such as adopting nest reuse, reflects the ability of birds to adapt to urban ecosystems (Saccavino et al., 2018). Several environmental pressures and factors can influence reproductive strategies, and the selection of reproductive strategies affects the survival, reproduction, and fitness of birds (Beardsell et al., 2016; Johnson & Pruett‐Jones, 2018).

### 4.2 Predation as a mechanism explaining nest reuse

Predation is a common cause of nest failure in birds, and other reasons for the failure include nest ectoparasites, bad weather, and human interference (Donahue et al., 2018). Although our study found a significant number of predation threats from aerial predators in urban habitats, the risk of urban predation was relatively low compared to rural or natural habitats (our findings in other comparative study of urban and rural Chinese blackbird, Zhang et al., 2024). Moreover, excellent permeability and ventilation ensure that breeding spotted dove reused nest in urban habitats are not at risk of large‐scale parasitic infections, which is supported by the fact that no breeding birds were detected to be parasitized during our investigation. Our results also indicated that reused nests have smaller twig volume and mean vimen diameter, which reduced the exposed area of nests (Table C: Appendix S1) and may offset the increased predation risk from nest reuse. This is consistent with the results of the predation risk analysis. No significant difference in the predation rate between reused and new nests was observed (*χ* ^2^ = 0.805, *df* = 1, *p* = .267). Contrarily, nest reuse by sparrowhawks, has been considered to increase predation risk. This is probably due to slightly later than average first egg‐laying and the use of open nests located two‐third of the way up trees without proper concealment (Otterbeck et al., 2019). Spotted doves build simple open‐cup nests (Zhao, 2001), and the nests are usually located in the tree canopy rather than on the main branches and concealed, which possibly makes the nests difficult for predators to find.

Taken together, we suggest that special nest structure is the material basis for the nest reuse in spotted doves and that the relatively low risk of predation in urban habitat and the scarcity of nest site resources due to urbanization increase the likelihood that birds prefer to reuse old nests.

### 4.3 Reproductive consequences of nesting behavior

Nest reuse as an adaptive behavior can improve avian reproductive success or fitness. In our study, reused nests had higher reproductive success rate (*χ* ^2^ = 8.461, *df* = 1, *p* = .004). In addition, reproductive success model supported that nest reuse was conducive to reproductive success (Table 1). However, in more urbanized areas, breeding durations are typically shorter for spotted doves (Figure C: Appendix S1), which is often caused by breeding failure due to ground predators and human disturbance in urban habitats that can adversely affect breeding success in birds. It is evident that nest reuse can increase breeding success to compensate for the adverse effects of urbanization to achieve improved fitness. Contrarily, other studies have also shown that the strategy of nest reuse does not have a positive effect on reproductive success (Cavitt et al., 1999; Koenig et al., 2021; Redmond et al., 2007). The main reason for this is that the nest material of these birds is relatively thicker and less permeable compared to spotted dove, and thus reused nests are more likely to carry disease or in vitro parasites (Rendell & Verbeek, 1996; Tomas et al., 2007; Wiebe, 2009). Furthermore, in contrast to the finding that reproductive success is positively correlated with nest size in most birds (Amininasab et al., 2022; Suarez et al., 2005), we found that small nest size favored successful breeding in spotted dove (Table B: Appendix S1), largely due to the fact that smaller clutch size (*n* = 2) avoided breeding failure due to small nest size and that less early‐stage energy expenditure leaves more energy expenditure for later breeding, thus increasing breeding success.

Birds have evolved appropriate breeding strategies to increase their fitness as a result of natural selection (Schneider & Griesser, 2015; Travers et al., 2010; Zanette et al., 2011), and urbanization has sped up this process (Corsini et al., 2021; Sepp et al., 2018). The preservation of old nest in urban habitats is crucial for the survival and reproduction of urban birds. Especially, in the process of urban greening, we should not arbitrarily destroy old nesting habitats. Moreover, we propose that future research should include numerous populations spanning the urban–rural gradient.

## AUTHOR CONTRIBUTIONS

**Yao Sheng:** Conceptualization (equal); data curation (equal); formal analysis (equal); writing – original draft (equal). **Mengjie Lu:** Supervision (equal); writing – review and editing (equal). **Junpeng Bai:** Software (equal); supervision (equal). **Xiaobin Xie:** Investigation (equal); methodology (equal). **Long Ma:** Formal analysis (equal). **Wanyou Li:** Supervision (equal). **Zhen Zhang:** Resources (equal). **Fang Ming:** Methodology (equal). **Xueli Zhang:** Data curation (equal). **Ziwei Zhang:** Project administration (equal). **Zhifeng Xu:** Supervision (equal). **Yuqing Han:** Project administration (equal). **Bicai Guan:** Visualization (equal). **Luzhang Ruan:** Writing – review and editing (equal).

## Supporting information


Appendix S1.

## Data Availability

The data that support the findings of this study are available in Figshare at https://doi.org/10.6084/m9.figshare.22787243.
