# Supplementary material for: Ecological drivers of nesting behavior in a subtropical city: An observational study on spotted doves
Source: Ecol Evol. 2024 Jul 3;14(7):e11655. doi: 10.1002/ece3.11655 (PMC11222170; doi:10.1002/ece3.11655)
Supplement: Supplementary file 1 — Appendix S1. [file ECE3-14-e11655-s001.docx]

**Appendix Table A** Description of ecological factors.

| Factors | Abbreviation | Description | Unit |
| --- | --- | --- | --- |
| Tree species | - | The species of trees that birds nested in | - |
| Tree height | TH | Height of nesting tree | m |
| Nest height | NH | Vertical distance from nest site to base of trunk | m |
| Diameter of the tree canopy | DTC | The mean of long and short diameters | m |
| Ground diameter | GD | The diameter around the base of the tree trunk | m |
| Branch support | BS | The sum of weighted average diameter of branches, weight is the ratio of the diameter of each branch to the sum of the diameters of all branches (only branches in direct contact with the nest were counted) | cm |
| Nest position | NP | The branch level of nesting position, if the nest located on the primary branch, we will record it with 1, on the secondary branch, record it with 2, and on the tertiary branch and above, record it with 3 | - |
| Distance from tree edge | DE | The nearest horizontal distance from the nest to tree crown edge | cm |
| Percent of canopy cover | PCC | Proportion of opaque part of crown, it was estimated proportion of opaque part by using photos which looking up at the crown | % |
| Percent of canopy cover over the nest | PCN | Proportion of opaque part of crown over the nest, it was estimated proportion of opaque part by using photos which looking up above the nest | % |
| Concealment | CC | The mean of concealment degree in four directions of the nest within 1m from the nest, it was estimated by using photos which include four directions of the nest within 1 m range (the percentage of whole the nest unexposed area in the photos was estimated, then, the mean values were calculated) | % |
| Distance from water | DW | Linear distance between nest and nearest water source | m |
| Small-scale urbanization score | SSUS | The first principal component of the variances (height of the highest building, percentages of building, vegetation, and impervious surfaces, distance from nest site to nighttime luminous source) is referred to as the small-scale urbanization score | - |
| Large-scale urbanization score | LSUS | The first principal component of the variances (percentages of building, vegetation, and impervious surfaces) is referred to as the large-scale urbanization score | - |

**Appendix Table B** Difference analysis of nest material parameters between reproductive success and reproductive failure of spotted dove.

|  | reproductive success | reproductive failure | | *t* | *df* | *P* |
| --- | --- | --- | --- | --- | --- | --- |
| Nest diameter | 16.967 ± 2.058 | | 17.881 ± 2.344 | -2.660 | 175 | **0.009** |
| Nest thickness | 6.026 ± 2.309 | | 6.468 ± 2.875 | -1.080 | 175 | 0.282 |
| Nest depth | 2.021 ± 0.915 | | 2.099 ± 0.875 | -0.567 | 175 | 0.572 |
| Mean of twig diameter | 2.297 ± 0.583 | | 2.270 ± 0.512 | 0.191 | 65 | 0.849 |
| Twig volume | 6654.802 ± 7376.353 | | 8794.916 ± 10925.343 | -0.866 | 71 | 0.389 |
| Mean of vimen diameter | 0.892 ± 0.201 | | 0.844 ± 0.161 | 1.106 | 71 | 0.273 |
| Vimen volume | 4389.081 ± 3157.105 | | 3499.139 ± 2238.112 | 1.389 | 71 | 0.169 |
| Total twig and vimen volume | 11043.883 ± 8128.834 | | 12294.055 ± 11030.791 | -0.493 | 71 | 0.624 |

Significant (*p* < 0.05) were highlighted in bold.

**Appendix Table C** Difference analysis of nest material parameters between nest reuse and nesting of spotted dove.

|  | Nest reuse | Nesting | |  | | *t* | *df* | | | *P* | |
| --- | --- | --- | --- | --- | --- | --- | --- | --- | --- | --- | --- |
| Nest diameter | 17.161 ± 2.187 | | 17.713 ± 2.306 | |  | -1.546 | | 175 | 0.124 | |  |
| Nest thickness | 5.692 ± 2.558 | | 6.617 ± 2.680 | |  | -2.227 | | 175 | **0.027** | |  |
| Nest depth | 1.968 ± 0.757 | | 2.123 ± 0.952 | |  | -1.183 | | 175 | 0.239 | |  |
| Mean of twig diameter | 2.170 ± 0.517 | | 2.357 ± 0.535 | |  | -1.397 | | 65 | 0.168 | |  |
| Twig volume | 5592.321 ± 6911.079 | | 10269.928 ± 11563.372 | |  | -2.059 | | 71 | **0.043** | |  |
| Mean of vimen diameter | 0.812 ± 0.207 | | 0.900 ± 0.130 | |  | -2.186 | | 71 | **0.032** | |  |
| Vimen volume | 3924.431 ± 3046.173 | | 3676.027 ± 2146.676 | |  | 0.407 | | 71 | 0.686 | |  |
| Total twig and vimen volume | 9516.753 ± 7517.834 | | 13945.955 ± 11657.499 | |  | -1.897 | | 71 | 0.062 | |  |

Significant (*p* < 0.05) were highlighted in bold.

**Appendix Table D** Pearson correlation analysis of ecological factors of spotted dove.

|  | NH | BS | NP | DE | PCC | CC | DW | TH | GD | DTC | PCN | SSUS | LSUS | REUSE |
| --- | --- | --- | --- | --- | --- | --- | --- | --- | --- | --- | --- | --- | --- | --- |
| NH | 1.000 |  |  |  |  |  |  |  |  |  |  |  |  |  |
| BS | 0.004 | 1.000 |  |  |  |  |  |  |  |  |  |  |  |  |
| NP | 0.241** | -0.155 | 1.000 |  |  |  |  |  |  |  |  |  |  |  |
| DE | 0.144* | 0.157** | 0.026 | 1.000 |  |  |  |  |  |  |  |  |  |  |
| PCC | -0.062 | -0.277 | 0.249** | -0.071 | 1.000 |  |  |  |  |  |  |  |  |  |
| CC | 0.011 | -0.067 | -0.155 | 0.105 | 0.275** | 1.000 |  |  |  |  |  |  |  |  |
| DW | -0.035 | -0.145 | 0.072 | -0.053 | 0.032 | 0.063 | 1.000 |  |  |  |  |  |  |  |
| TH | **0.891**** | 0.082 | 0.130* | 0.304** | -0.125 | 0.009 | -0.046 | 1.000 |  |  |  |  |  |  |
| GD | **0.626**** | 0.246** | 0.129* | 0.207** | -0.115 | -0.020 | -0.162 | **0.622**** | 1.000 |  |  |  |  |  |
| DTC | **0.511**** | -0.084 | 0.298** | 0.220** | 0.135* | -0.014 | 0.000 | **0.536**** | 0.269** | 1.000 |  |  |  |  |
| PCN | -0.055 | -0.148 | 0.081 | -0.030 | **0.558**** | 0.274** | 0.039 | -0.074 | -0.086 | -0.030 | 1.000 |  |  |  |
| SSUS | 0.076 | -0.159 | 0.198** | 0.068 | 0.213** | 0.030 | 0.156** | 0.131* | -0.073 | 0.171** | 0.121* | 1.000 |  |  |
| LSUS | 0.168** | -0.027 | 0.062 | 0.167** | -0.017 | 0.089 | 0.188** | 0.195** | 0.098 | 0.105 | -0.029 | 0.423** | 1.000 |  |
| REUSE | 0.072 | 0.068 | -0.104 | 0.134* | -0.031 | 0.077 | -0.084 | 0.090 | 0.077 | -0.023 | 0.021 | -0.028 | 0.134* | 1.000 |

*|r|* > 0.500 are shown in bold. * (*p* < 0.050), ** (*p* < 0.010). Variable descriptions are found in Appendix Table A.

**Appendix Table E** Pearson correlation analysis of ecological factors of spotted dove monitored by infrared tracking cameras.

|  | NH | PCC | DTC | BS | NP | DE | NCC | CC | DW | GD | TH | SSUS | LSUS | REUSE |
| --- | --- | --- | --- | --- | --- | --- | --- | --- | --- | --- | --- | --- | --- | --- |
| NH | 1.000 |  |  |  |  |  |  |  |  |  |  |  |  |  |
| PCC | -0.154 | 1.000 |  |  |  |  |  |  |  |  |  |  |  |  |
| DTC | 0.345** | 0.075 | 1.000 |  |  |  |  |  |  |  |  |  |  |  |
| BS | -0.034 | -0.238 | -0.326 | 1.000 |  |  |  |  |  |  |  |  |  |  |
| NP | 0.226* | 0.246* | 0.418** | -0.410 | 1.000 |  |  |  |  |  |  |  |  |  |
| DE | 0.157 | 0.050 | 0.319** | 0.040 | 0.107 | 1.000 |  |  |  |  |  |  |  |  |
| NCC | 0.056 | **0.512**** | 0.009 | 0.025 | 0.211* | 0.137 | 1.000 |  |  |  |  |  |  |  |
| CC | -0.096 | 0.363** | 0.005 | -0.104 | -0.117 | 0.142 | 0.297** | 1.000 |  |  |  |  |  |  |
| DW | -0.012 | -0.017 | -0.037 | -0.178 | 0.091 | -0.165 | 0.022 | -0.012 | 1.000 |  |  |  |  |  |
| GD | **0.677**** | -0.166 | 0.114 | 0.165 | 0.189 | 0.263* | 0.053 | -0.063 | -0.059 | 1.000 |  |  |  |  |
| TH | **0.791**** | -0.231 | 0.422** | 0.055 | 0.091 | 0.196 | 0.046 | -0.097 | -0.043 | **0.617**** | 1.000 |  |  |  |
| SSUS | -0.074 | 0.202 | 0.199 | -0.241 | 0.136 | 0.060 | -0.016 | -0.002 | 0.134 | 0.087 | 0.066 | 1.000 |  |  |
| LSUS | 0.084 | -0.097 | 0.202 | -0.142 | 0.132 | 0.181 | -0.037 | 0.074 | 0.163 | 0.206 | 0.216* | 0.379** | 1.000 |  |
| REUSE | 0.160 | -0.106 | -0.249 | 0.099 | -0.194 | -0.027 | 0.078 | 0.080 | -0.083 | 0.141 | 0.136 | -0.184 | 0.107 | 1.000 |

*|r|* > 0.500 are shown in bold. * (*p* < 0.050), ** (*p* < 0.010). Variable descriptions are found in Appendix Table A.

**Appendix Table F** Home range and flight distance of spotted dove.

| Satellite tracker ID | Capture year | Duration (day) | Home range (m^2^) | Maximum flight distance (m) | Minimum flight distance (m) |
| --- | --- | --- | --- | --- | --- |
| F202194 | 2021 | 201 | 91145.80 | 421.23 | 0.56 |
| F202299 | 2021 | 115 | 33832.40 | 144.12 | 0.22 |
| 4054 | 2019 | 32 | 15265.30 | 190.05 | 1.00 |
| F202285 | 2021 | 48 | 45626.30 | 321.16 | 0.89 |
| Mean ± SD |  |  | 46467.45 ± 32301.09 | 269.14 ± 126.13 | 0.67 ± 0.35 |

**Appendix Table G** Principal component analysis of small-scale and large-scale urbanization characteristics variables.

|  | Component (small scale) |  | Component (large scale) |  |
| --- | --- | --- | --- | --- |
|  | PC1 | PC2 | PC1 | PC2 |
| Eigenvalue | 34.313 | 14.182 | 17.517 | 6.496 |
| Percentage of variance (%) | 79.850 | 13.640 | 83.950 | 11.540 |
| Cumulative percentage of variance (%) | 79.850 | 93.490 | 83.950 | 95.500 |
| Correlation of components to nest site factors |  |  |  |  |
| Building percentage | 0.427 | **-0.894** | 0.380 | **0.708** |
| Vegetation percentage | **-0.623** | -0.330 | -0.583 | **0.683** |
| Impervious surfaces percentage | **0.648** | 0.287 | **0.718** | 0.179 |
| Height of the highest building | 0.045 | -0.072 | - | - |
| Distance from the nest site to nighttime luminous source | -0.090 | 0.064 | - | - |

Loading of variable with absolute value > 0.6 are marked in bold.

**Appendix Table H** The *t*-test analysis of ecological factors for reproductive or reuse state of spotted dove.

| Reproductive state (Mean ± SD) | | | | | Reuse state (Mean ± SD) | | | |
| --- | --- | --- | --- | --- | --- | --- | --- | --- |
| Factors | Reproductive success (n_1_ = 136) | reproductive  failure (n_2_ = 166) | *t* | *p* | nest reuse  (n_1_ = 115) | nesting  (n_2_ = 187) | *t* | *p* |
| tree height | 7.792 ± 2.375 | 6.595 ± 2.186 | 4.551 | **<0.001** | 7.401 ± 2.212 | 6.970 ± 2.416 | 1.557 | 0.121 |
| nest height | 6.283 ± 2.053 | 5.097 ± 1.962 | 5.118 | **<0.001** | 5.823 ± 1.940 | 5.513 ± 2.167 | 1.253 | 0.211 |
| ground diameter | 27.113 ± 11.455 | 22.540 ± 10.216 | 3.664 | **<0.001** | 25.686 ± 11.091 | 23.931 ± 10.938 | 1.346 | 0.179 |
| diameter of the tree canopy | 5.399 ± 2.045 | 4.985 ± 1.758 | 1.862 | 0.064 | 5.116 ± 2.018 | 5.205 ± 1.829 | -0.392 | 0.695 |
| distance from tree edge | 98.571 ± 94.557 | 86.565 ± 65.605 | 1.254 | 0.211 | 105.577 ± 94.316 | 83.604 ± 68.764 | 2.169 | **0.031** |
| branch support | 16.101 ±23.234 | 15.499 ±26.179 | 0.209 | 0.834 | 17.935 ± 27.340 | 14.438 ± 23.176 | 1.188 | 0.386 |
| nest position | 2.735 ±0.505 | 2.608 ±0.601 | 1.993 | **0.047** | 2.591 ± 0.560 | 2.711 ± 0.560 | -1.806 | 0.072 |
| percent of canopy cover | 81.360 ± 9.805 | 76.494 ± 15.872 | 3.263 | **0.001** | 78.148 ± 12.939 | 79.016 ± 14.137 | -0.535 | 0.593 |
| percent of canopy cover over the nest | 77.728 ± 14.882 | 75.687 ± 18.714 | 1.056 | 0.292 | 77.061 ± 16.284 | 76.326 ± 17.617 | 0.362 | 0.718 |
| concealment | 74.950 ± 15.869 | 73.720 ± 20.218 | 0.593 | 0.554 | 76.078 ± 17.596 | 73.160 ± 18.787 | 1.342 | 0.181 |
| distance to water sources | 173.059 ± 121.011 | 178.328 ± 124.970 | -0.370 | 0.712 | 162.799 ± 118.608 | 184.046 ± 125.295 | -1.460 | 0.145 |
| small-scale  urbanization score | -1.807 ± 36.278 | 1.480 ± 32.652 | -0.828 | 0.408 | -1.231 ± 34.324 | 0.757 ± 34.377 | -0.488 | 0.626 |
| large-scale  urbanization score | -0.462 ± 18.868 | 0. 378 ± 16.375 | -0.414 | 0.679 | 2.989 ± 19.560 | -1.838 ± 15.910 | 2.343 | **0.020** |

Significant results (*p* < 0.05) were highlighted in bold. Variable descriptions are found in Appendix Table A.

**Appendix Fig. A.** Pictures of (a) reproductive failure and (b) reproductive success nests.


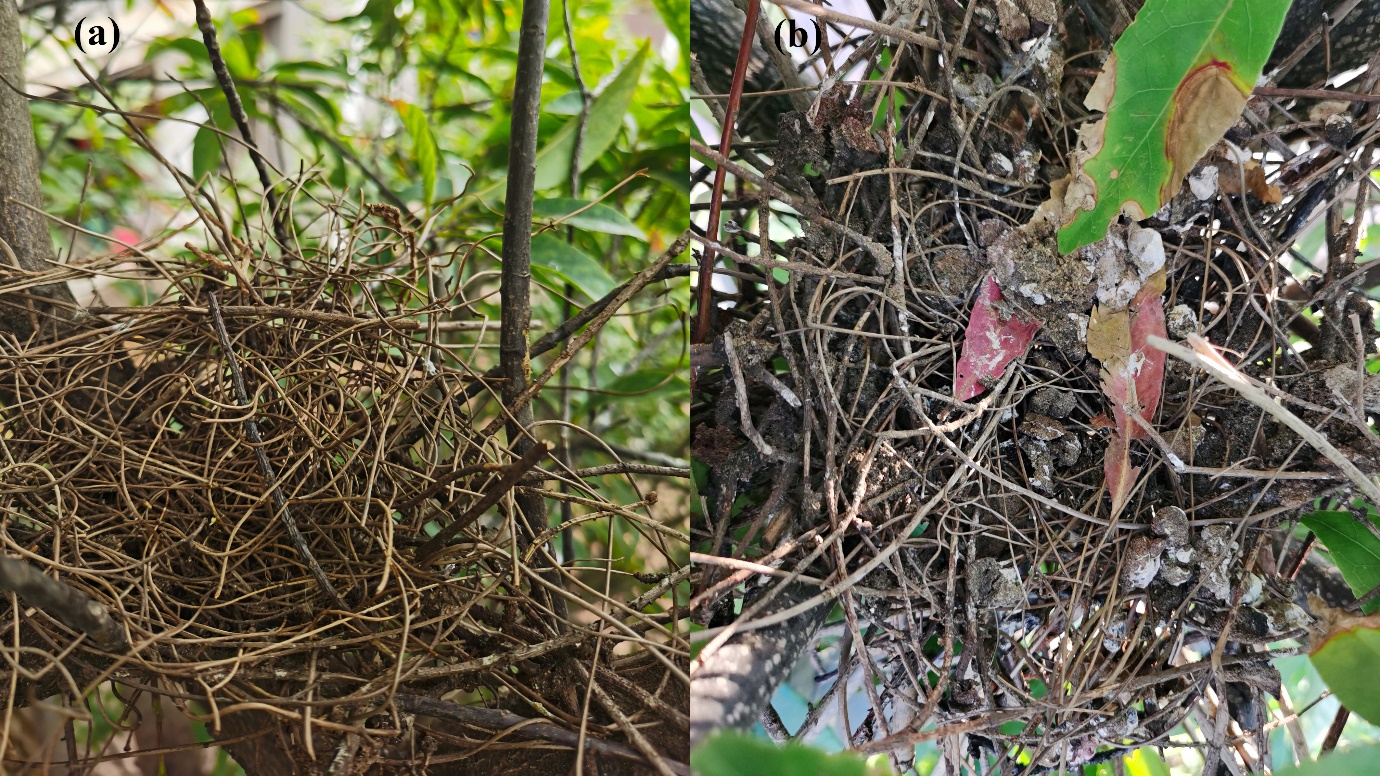


**Appendix Fig. B.** Correlation between reproductive duration and best model parameters of the spotted dove. Model fit, 95% confidence intervals, and *p*-values are shown. Variables descriptions are found in Appendix Table A.





**Appendix Fig. C.** Effect of large-scale urbanization score on the coloration of reproductive duration of the spotted dove. Model fit, 95% confidence intervals, and *p*-values are shown. Variables descriptions are found in Appendix Table A.
